# The crystal structures of four di­meth­oxy­benzaldehyde isomers

**DOI:** 10.1107/S2056989018017152

**Published:** 2019-01-01

**Authors:** Sander J. T. Brugman, Anthonius H. J. Engwerda, Emma Kalkman, Erik de Ronde, Paul Tinnemans, Elias Vlieg

**Affiliations:** a Radboud University, Institute for Molecules and Materials, Heyendaalseweg 135, 6525 AJ Nijmegen, The Netherlands

**Keywords:** crystal structure, di­meth­oxy­benzaldehyde, polymorphism

## Abstract

The crystal structures of four di­meth­oxy­benzaldehyde (C_9_H_10_O_3_) isomers, namely the 2,3-, 2,4-, 2,5- and 3,5- isomers, are reported and compared to the previously reported crystal structures of 3,4-di­meth­oxy­benzaldehyde and 2,6-di­meth­oxy­benzaldehyde. All di­meth­oxy­benzaldehyde mol­ecules in the crystal structures are nearly planar. The largest deviation (1.2 Å) from the aromatic plane is found for one of the meth­oxy groups of 2,3-di­meth­oxy­benzaldehyde. Upon rapid cooling of 3,4-di­meth­oxy­benzaldehyde and 3,5-di­meth­oxy­benzaldehyde, a metastable polymorph is formed. The crystal studied for the 3,5- isomer was refined as a two-component twin.

## Abstract 2

The crystal structures of four di­meth­oxy­benzaldehyde (C_9_H_10_O_3_) isomers are reported and compared to the previously reported crystal structures of 3,4-di­meth­oxy­benzaldehyde and 2,6-di­meth­oxy­benzaldehyde. All di­meth­oxy­benzaldehyde mol­ecules in the crystal structure are nearly planar.

## Chemical context   

Di­meth­oxy­benzaldehydes (DMBz) are often used as starting materials in condensation reactions forming Schiff base compounds. Schiff base compounds are versatile ligands in numerous metal–organic complexes that are used as a catalyst. Examples include C—O coupling reactions (Maity *et al.*, 2015 ▸), the Suzuiki–Miyaura reaction (Das & Linert, 2016 ▸), nitro­aldol reactions (Handa *et al.*, 2008 ▸) and a wide variety of other reactions (Gupta & Sutar, 2008 ▸).
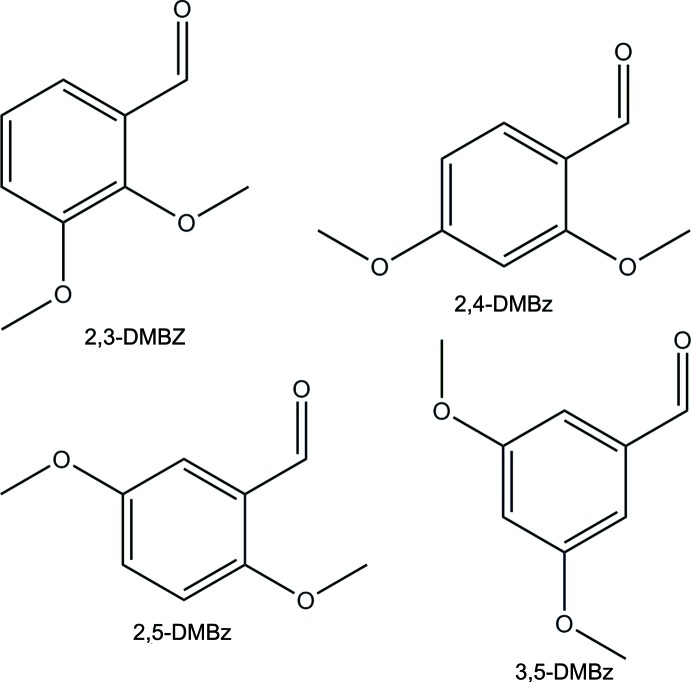


**Figure 1:**
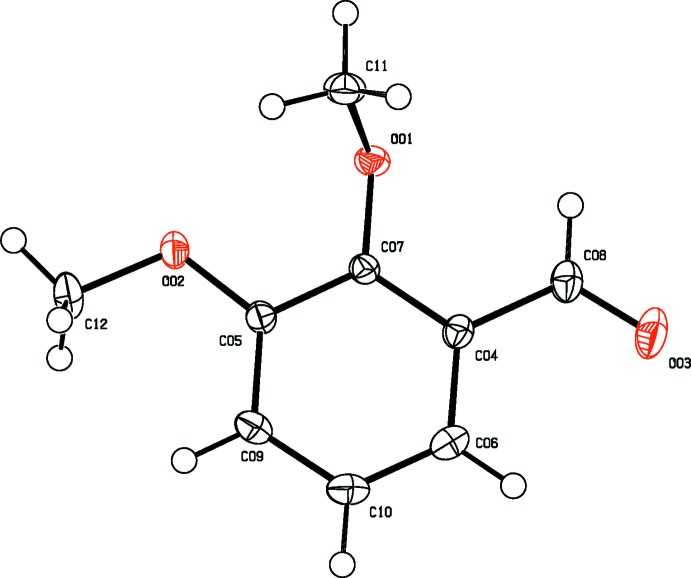
The mol­ecular structure of 2,3-DMBz, showing displacement ellipsoids drawn at the 50% probability level.

**Figure 2:**
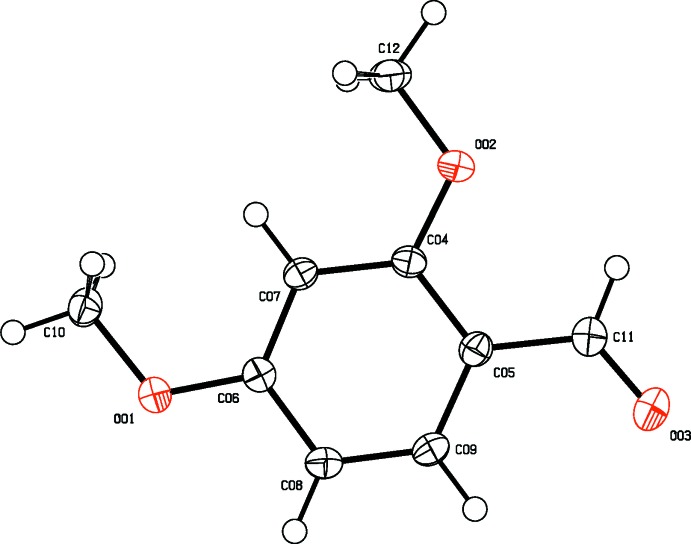
The mol­ecular structure of 2,4-DMBz, showing displacement ellipsoids drawn at the 50% probability level.

**Figure 3:**
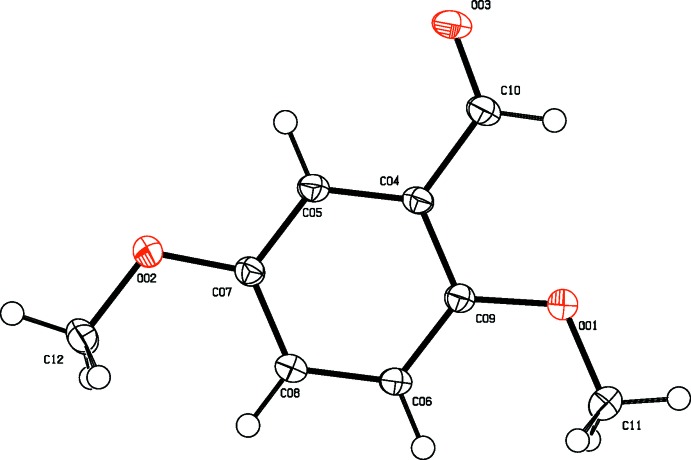
The mol­ecular structure of 2,5-DMBz, showing displacement ellipsoids drawn at the 50% probability level.

**Figure 4:**
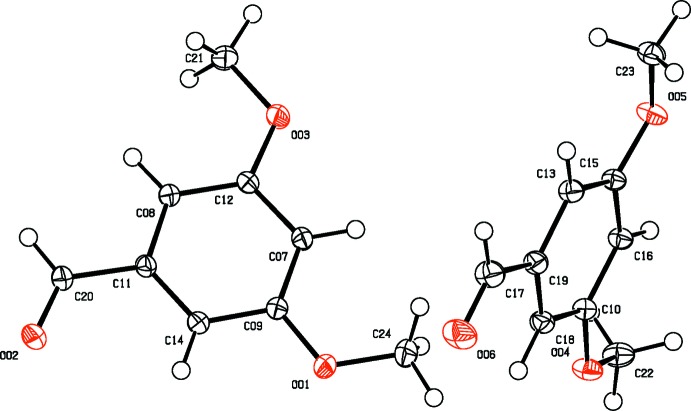
The mol­ecular structure of 3,5-DMBz, showing displacement ellipsoids drawn at the 50% probability level.

Whereas the crystal structures of nearly 100 DMBz derivatives have been published, not all of the crystal structures of the DMBz starting compounds are known. Only the crystal structures of 3,4-DMBz (de Ronde *et al.*, 2016 ▸) and 2,6-DMBz (Lemercier *et al.*, 2014 ▸) have been reported. In this work, we report the structures of the four other di­meth­oxy­benzaldehyde isomers, namely 2,3-DMBz (Fig. 1 ▸), 2,4-DMBz (Fig. 2 ▸), 2,5-DMBz (Fig. 3 ▸) and 3,5-DMBz (Fig. 4 ▸).

## Structural commentary   

All four reported isomers crystallize in the monoclinic space group *P*2_1_/*c*, which is also the case for the previously reported 2,6-DMBz (Lemercier *et al.*, 2014 ▸). On the other hand, 3,4-DMBz was reported to crystallize in space group *Pna*2_1_ (de Ronde *et al.*, 2016 ▸). 3,5-DMBz has two mol­ecules in the asymmetric unit, while the other crystal structures have one mol­ecule in the asymmetric unit. The DMBz mol­ecules in the crystal structures are almost planar (Table 1 ▸). The biggest deviation is found in the 2,3-DMBz in which one of the meth­oxy groups deviates by 1.2 Å from the aromatic plane.

## Supra­molecular features   

**Figure 5:**
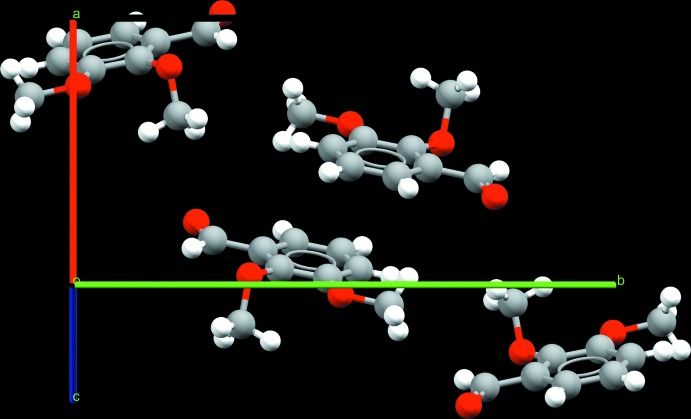
Crystal structure of 2,3-DMBz showing the orientation of the meth­oxy groups. One of the meth­oxy groups lies in the plane of the aromatic ring. The second meth­oxy group points towards the aldehyde group of a neighbouring 2,3-DMBz mol­ecule.

**Figure 6:**
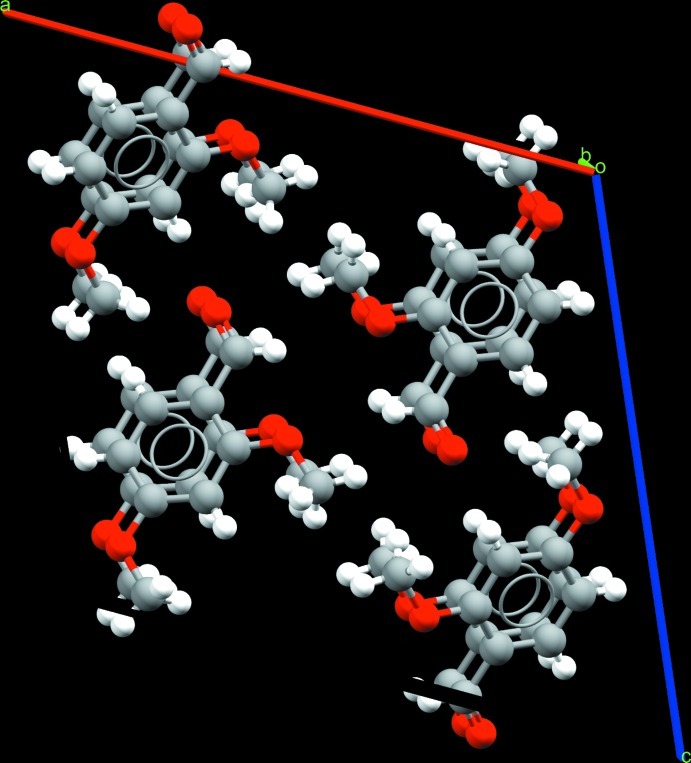
A view along the *b* axis of the crystal structure of 2,4-DMBz, in which π–π stacking inter­actions between the aromatic rings are present.

**Figure 7:**
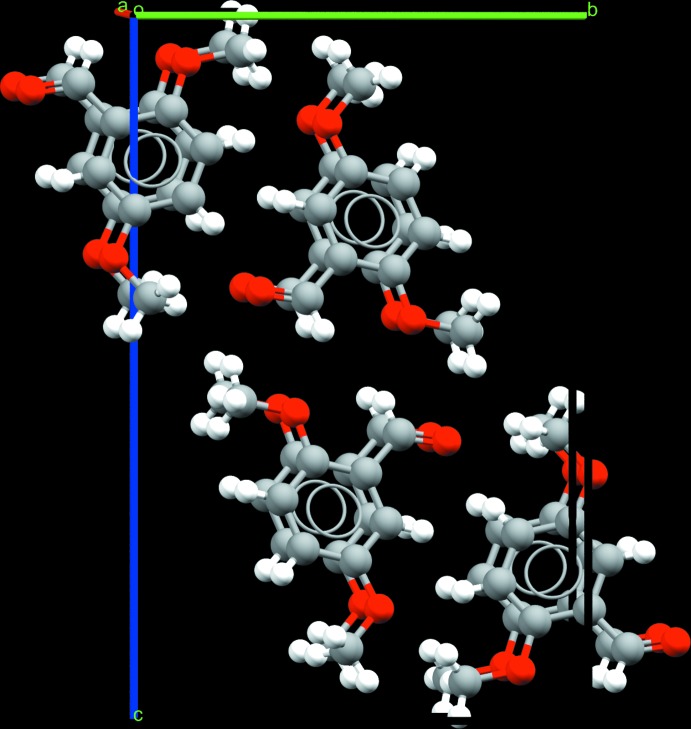
A view along the *a* axis of the crystal structure of 2,5-DMBz, in which π–π stacking inter­actions between the aromatic rings are present.

In the crystal structure of 2,3-DMBz, one of the meth­oxy groups lies in the plane of the aromatic ring (see Fig. 5 ▸). The second meth­oxy group points towards the aldehyde group of a neighboring 2,3-DMBz mol­ecule. In the crystal structure of 2,4-DMBz, shown in Fig. 6 ▸, π–π stacking inter­actions between the aromatic rings are present along the *b*-axis direction [centroid–centroid separation = 3.9638 (2) Å]. Similarly, in the crystal structure of 2,5-DMBz, aromatic π–π stacking inter­actions are present along the *a*-axis direction [centroid–centroid separation = 3.8780 (3) Å], as shown in Fig. 7 ▸. The crystal structures of 2,6-DMBz (Lemercier *et al.*, 2014 ▸), 3,4-DMBz (de Ronde *et al.*, 2016 ▸) and 3,5-DMBz do not exhibit aromatic π–π stacking inter­actions. As mentioned above, only 3,5-DMBz has two mol­ecules in the asymmetric unit, whereas the other crystal structures have one mol­ecule in the asymmetric unit.

## Polymorphism   

**Figure 8:**
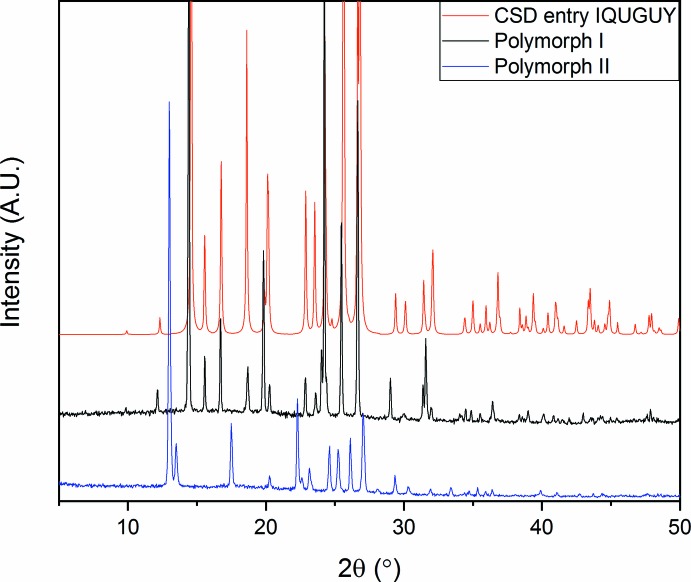
Powder X-ray diffraction measurements of form I (black) and II (blue) of 3,4-DMBz. The powder pattern (red) was calculated from the crystal structure by de Ronde *et al.* (2016 ▸).

**Figure 9:**
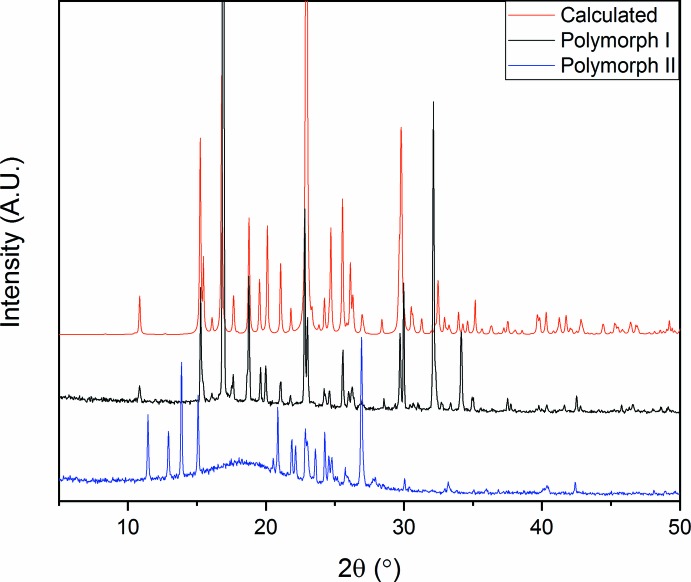
Powder X-ray diffraction measurements of form I (black) and II (blue) of 3,5-DMBz. The powder pattern (red) was calculated from the crystal structure.

Polymorph screening using differential scanning calorimetry did not reveal any phase transitions for any DMBz between 133 K and the melting point of the compound (Table 2 ▸). On the other hand, a metastable polymorphic form was discovered after rapidly cooling from the melt for both 3,4-DMBz for which the crystal structure was reported previously (de Ronde *et al.* 2016 ▸) and 3,5-DMBz. In the course of hours, these polymorphic forms transformed into the stable forms. Powder X-ray diffraction measurements confirmed the existence of these metastable forms (3,4-DMBz: Figs. 8 ▸, 3 ▸, 5 ▸-DMBz: Fig. 9 ▸).

## Database survey   

A search in the Cambridge Structural Database (Version 5.39, update February 2018, Groom *et al.*, 2016 ▸) for di­meth­oxy­benzaldehydes derivatives yielded the crystal structure of 93 compounds, which can be subdivided into fourteen 2,3-DMBz derivatives (including two solvates), fifteen 2,4-DMBz deriv­atives (including four solvates), ten 2,5-DMBz derivatives (including two solvates), nine 2,6-DMBz derivatives (including one solvate), forty two 3,4-DMBz derivatives (including nine solvates) and three 3,5-DMBz derivatives.

## Synthesis and crystallization   

### 2,3-di­meth­oxy­benzaldehyde   

30 mg of 2,3-di­meth­oxy­benzaldehyde (97%, Fluoro­chem) was dissolved in 4 mL of isopropyl ether. Slow evaporation of a 1:1 mixture of this solution and heptane yielded colorless block-shaped crystals suitable for single crystal X-ray diffraction.

### 2,4-di­meth­oxy­benzaldehyde   

25 mg of 2,4-di­meth­oxy­benzaldeyhyde (98%, Aldrich) was dissolved in a 1:1 ratio of hepta­ne/acetone (1.5 mL). Slow evaporation yielded colorless block-shaped crystals suitable for single crystal X-ray diffraction.

### 2,5-di­meth­oxy­benzaldehyde   

1 g of 2,5-di­meth­oxy­benzaldeyhyde (97%, Acros Organics) was dissolved in a mixture of heptane (1 mL) and acetone (1 mL). Slow evaporation yielded colorless needles suitable for single crystal X-ray diffraction.

### 3,5-di­meth­oxy­benzaldehyde   

It was noted that 3,5-di­meth­oxy­benzaldehyde (98%, Aldrich) oils out from solution, therefore the same method was used as had previously been employed for 3,4-di­meth­oxy­benzaldehyde (de Ronde *et al.*, 2016 ▸). In short, a few crystals of the commercial powder were added to a saturated solution in water. Subsequently, the temperature was cycled between 298 and 303 K. This resulted in the growth of single crystals suitable for single-crystal X-ray diffraction in several weeks.

## Refinement   

Crystal data, data collection and structure refinement details are summarized in Table 3 ▸. H atoms were positioned geom­etrically and refined as riding with C—H = 0.95–0.96 and *U*_iso_(H) = 1.2–1.5*U*_eq_(C). The crystal of 3,5-DMBz studied was refined as a two-component twin.

## Supplementary Material

Crystal structure: contains datablock(s) 2,3DMBz, 2,4DMBz, 2,5DMBz, 3,5DMBz. DOI: 10.1107/S2056989018017152/zp2031sup1.cif

Structure factors: contains datablock(s) 2,3DMBz. DOI: 10.1107/S2056989018017152/zp203123DMBzsup2.hkl

Structure factors: contains datablock(s) 2,4DMBz. DOI: 10.1107/S2056989018017152/zp203124DMBzsup3.hkl

Structure factors: contains datablock(s) 2,5DMBz. DOI: 10.1107/S2056989018017152/zp203125DMBzsup4.hkl

Structure factors: contains datablock(s) 3,5DMBz. DOI: 10.1107/S2056989018017152/zp203135DMBzsup5.hkl

Supporting information file. DOI: 10.1107/S2056989018017152/zp203123DMBzsup6.cml

Supporting information file. DOI: 10.1107/S2056989018017152/zp203124DMBzsup7.cml

Supporting information file. DOI: 10.1107/S2056989018017152/zp203125DMBzsup8.cml

Supporting information file. DOI: 10.1107/S2056989018017152/zp203135DMBzsup9.cml

CCDC references: 1882761, 1882760, 1882759, 1882758

Additional supporting information:  crystallographic information; 3D view; checkCIF report

## Figures and Tables

**Table 1:** Deviation from the aromatic plane (in Å)

	2,3-DMBz	2,4-DMBz	2,5-DMBz	2,6-DMBz (CSD refcode: LIZLAJ)	3,4-DMBz (CSD refcode: IQUGUY)	3,5-DMBz (mol­ecule 1)	3,5-DMBz (mol­ecule 2)
Aldehyde C	0.020	0.060	0.004	0.027	0.020	0.027	0.022
Aldehyde O	0.104	0.089	0.113	0.015	0.095	0.019	0.047
Meth­oxy 1 O	0.048	0.013	0.033	0.011	0.002	0.009	0.015
Meth­oxy 1 C	1.200	0.122	0.099	0.017	0.001	0.087	0.258
Meth­oxy 2 O	0.035	0.019	0.025	0.024	0.033	0.013	0.019
Meth­oxy 2 C	0.013	0.074	0.109	0.040	0.337	0.020	0.109

Meth­oxy 1 and 2 are defined in the same order as the atomic labels, as shown in Fig. 4 ▸.

**Table 2:** Melting point (in K) of DMBz as determined using the onset temperature of differential scanning calorimetry

	2,3-DMBz	2,4-DMBz	2,5-DMBz	2,6-DMBz	3,4-DMBz	3,5-DMBz
Polymorph I (stable form)	322	341	321	368	317	319
Polymorph II					*	310

* Melting point could not be determined using differential scanning calorimetry.

**Table 3:** Experimental details

	2,3DMBz	2,4DMBz	2,5DMBz	3,5DMBz
Crystal data				
Chemical formula	C_9_H_10_O_3_	C_9_H_10_O_3_	C_9_H_10_O_3_	C_9_H_10_O_3_
*M*_r_	166.17	166.17	166.17	166.17
Crystal system, space group	Monoclinic, *P*2_1_/*c*	Monoclinic, *P*2_1_/*c*	Monoclinic, *P*2_1_/*n*	Monoclinic, *P*2_1_/*c*
Temperature (K)	150	150	150	150
*a*, *b*, *c* (Å)	7.6152 (3), 15.5513 (6), 7.5891 (3)	15.1575 (8), 3.9638 (2), 14.6181 (8)	3.8780 (3), 11.5513 (7), 17.8153 (12)	11.7602 (5), 13.8957 (6), 11.4352 (5)
β (°)	115.8831 (18)	113.8388 (19)	91.808 (2)	118.642 (2)
*V* (Å^3^)	808.59 (6)	803.35 (7)	797.66 (10)	1640.03 (13)
*Z*	4	4	4	8
Radiation type	Mo *K*α	Mo *K*α	Mo *K*α	Mo *K*α
μ (mm^−1^)	0.10	0.10	0.10	0.10
Crystal size (mm)	0.49 × 0.45 × 0.16	0.50 × 0.43 × 0.40	0.74 × 0.38 × 0.13	0.50 × 0.43 × 0.40

Data collection				
Diffractometer	Bruker D8 Quest *APEX3*	Bruker D8 Quest *APEX3*	Bruker D8 Quest *APEX3*	Bruker D8 Quest *APEX3*
Absorption correction	Multi-scan (*SADABS*; Krause et al., 2015 ▸)	Multi-scan (*SADABS*; Krause et al., 2015 ▸)	Multi-scan (*SADABS*; Krause et al., 2015 ▸)	Multi-scan (*SADABS*; Krause et al., 2015 ▸)
*T*_min_, *T*_max_	0.672, 0.747	0.685, 0.746	0.705, 0.747	0.703, 0.747
No. of measured, independent and observed [*I* > 2σ(*I*)] reflections	17821, 4126, 3160	15236, 2461, 2171	30235, 3873, 3276	53075, 7976, 6730
*R*_int_	0.032	0.020	0.024	0.030
(sin θ/λ)_max_ (Å^−1^)	0.849	0.714	0.834	0.836

Refinement				
*R*[*F* ^2^ > 2σ(*F* ^2^)], *wR*(*F* ^2^), *S*	0.043, 0.130, 1.02	0.039, 0.117, 1.03	0.039, 0.124, 1.02	0.042, 0.126, 1.05
No. of reflections	4126	2461	3873	7976
No. of parameters	111	111	111	222
H-atom treatment	H-atom parameters constrained	H-atom parameters constrained	H-atom parameters constrained	H-atom parameters constrained
Δρ_max_, Δρ_min_ (e Å^−3^)	0.60, −0.24	0.40, −0.24	0.54, −0.22	0.48, −0.26

Computer programs: *APEX3* and *SAINT* (Bruker, 2012 ▸), *PEAKREF* (Schreurs, 2013 ▸), *SHELXT2014/4* (Sheldrick, 2015*a* ▸), *SHELXL2016/6* (Sheldrick, 2015*b* ▸), *PLATON* (Spek, 2009 ▸) and *ShelXLe* (Hübschle *et al.*, 2011 ▸).

## supplementary crystallographic information

### 2,3-Dimethoxybenzaldehyde (23DMBz) Crystal data

**Table:**

C_9_H_10_O_3_	*D*_x_ = 1.365 Mg m^−^^3^
*M**_r_* = 166.17	Melting point: 322 K
Monoclinic, *P*2_1_/*c*	Mo *K*α radiation, λ = 0.71073 Å
*a* = 7.6152 (3) Å	Cell parameters from 6893 reflections
*b* = 15.5513 (6) Å	θ = 2.6–36.9°
*c* = 7.5891 (3) Å	µ = 0.10 mm^−^^1^
β = 115.8831 (18)°	*T* = 150 K
*V* = 808.59 (6) Å^3^	Block, colourless
*Z* = 4	0.49 × 0.45 × 0.16 mm
*F*(000) = 352	

### 2,3-Dimethoxybenzaldehyde (23DMBz) Data collection

**Table:**

Bruker D8 Quest APEX3 diffractometer	4126 independent reflections
Radiation source: sealed tube	3160 reflections with *I* > 2σ(*I*)
Graphite monochromator	*R*_int_ = 0.032
Detector resolution: 10.4 pixels mm^-1^	θ_max_ = 37.1°, θ_min_ = 2.6°
φ and ω scans	*h* = −12→12
Absorption correction: multi-scan (SADABS; Krause et al., 2015)	*k* = −26→26
*T*_min_ = 0.672, *T*_max_ = 0.747	*l* = −12→12
17821 measured reflections	

### 2,3-Dimethoxybenzaldehyde (23DMBz) Refinement

**Table:**

Refinement on *F*^2^	Primary atom site location: structure-invariant direct methods
Least-squares matrix: full	Secondary atom site location: difference Fourier map
*R*[*F*^2^ > 2σ(*F*^2^)] = 0.043	Hydrogen site location: inferred from neighbouring sites
*wR*(*F*^2^) = 0.130	H-atom parameters constrained
*S* = 1.02	*w* = 1/[σ^2^(*F*_o_^2^) + (0.0743*P*)^2^ + 0.0948*P*] where *P* = (*F*_o_^2^ + 2*F*_c_^2^)/3
4126 reflections	(Δ/σ)_max_ = 0.001
111 parameters	Δρ_max_ = 0.60 e Å^−^^3^
0 restraints	Δρ_min_ = −0.24 e Å^−^^3^

### 2,3-Dimethoxybenzaldehyde (23DMBz) Special details

**Table:**

Geometry. All esds (except the esd in the dihedral angle between two l.s. planes) are estimated using the full covariance matrix. The cell esds are taken into account individually in the estimation of esds in distances, angles and torsion angles; correlations between esds in cell parameters are only used when they are defined by crystal symmetry. An approximate (isotropic) treatment of cell esds is used for estimating esds involving l.s. planes.

### 2,3-Dimethoxybenzaldehyde (23DMBz) Fractional atomic coordinates and isotropic or equivalent isotropic displacement parameters (Å^2^)

**Table:**

	*x*	*y*	*z*	*U*_iso_*/*U*_eq_	
O01	0.84423 (8)	0.67610 (3)	0.72112 (7)	0.01819 (11)	
O02	0.93597 (9)	0.50880 (4)	0.76827 (8)	0.02194 (12)	
O03	0.42554 (9)	0.77393 (5)	0.22027 (10)	0.03084 (15)	
C04	0.60779 (10)	0.65162 (5)	0.38944 (10)	0.01633 (12)	
C05	0.79724 (10)	0.53265 (4)	0.58899 (9)	0.01586 (12)	
C06	0.50903 (11)	0.59449 (5)	0.23432 (10)	0.02083 (14)	
H06	0.410967	0.615175	0.113675	0.025*	
C07	0.75191 (9)	0.62098 (4)	0.56613 (9)	0.01466 (12)	
C08	0.55760 (11)	0.74397 (5)	0.36618 (11)	0.02121 (14)	
H08	0.632072	0.781899	0.470279	0.025*	
C09	0.69865 (11)	0.47667 (5)	0.43414 (11)	0.01968 (13)	
H09	0.729027	0.417067	0.448365	0.024*	
C10	0.55498 (11)	0.50806 (5)	0.25774 (11)	0.02239 (15)	
H10	0.487993	0.469473	0.152498	0.027*	
C11	1.04331 (11)	0.69266 (5)	0.75867 (12)	0.02355 (15)	
H11A	1.113534	0.638043	0.778082	0.035*	
H11B	1.106109	0.728092	0.876679	0.035*	
H11C	1.045946	0.723144	0.646820	0.035*	
C12	0.98881 (13)	0.41998 (5)	0.79585 (12)	0.02495 (16)	
H12A	0.872738	0.385147	0.769517	0.037*	
H12B	1.086092	0.410593	0.931161	0.037*	
H12C	1.043851	0.403253	0.705808	0.037*	

### 2,3-Dimethoxybenzaldehyde (23DMBz) Atomic displacement parameters (Å^2^)

**Table:**

	*U*^11^	*U*^22^	*U*^33^	*U*^12^	*U*^13^	*U*^23^
O01	0.0179 (2)	0.0172 (2)	0.0179 (2)	−0.00017 (17)	0.00634 (17)	−0.00429 (17)
O02	0.0282 (3)	0.0151 (2)	0.0174 (2)	0.00471 (19)	0.0052 (2)	0.00247 (17)
O03	0.0228 (3)	0.0306 (3)	0.0343 (3)	0.0108 (2)	0.0081 (2)	0.0124 (3)
C04	0.0142 (3)	0.0176 (3)	0.0168 (3)	0.0012 (2)	0.0064 (2)	0.0019 (2)
C05	0.0180 (3)	0.0140 (3)	0.0157 (2)	0.0007 (2)	0.0075 (2)	0.0007 (2)
C06	0.0171 (3)	0.0265 (4)	0.0164 (3)	−0.0016 (2)	0.0049 (2)	−0.0001 (2)
C07	0.0147 (3)	0.0138 (3)	0.0153 (2)	0.0003 (2)	0.0064 (2)	−0.00084 (19)
C08	0.0185 (3)	0.0207 (3)	0.0250 (3)	0.0049 (2)	0.0100 (3)	0.0050 (3)
C09	0.0240 (3)	0.0156 (3)	0.0207 (3)	−0.0029 (2)	0.0110 (3)	−0.0034 (2)
C10	0.0229 (3)	0.0240 (3)	0.0189 (3)	−0.0061 (3)	0.0078 (3)	−0.0057 (2)
C11	0.0193 (3)	0.0225 (3)	0.0254 (3)	−0.0045 (3)	0.0067 (3)	−0.0055 (3)
C12	0.0324 (4)	0.0170 (3)	0.0272 (3)	0.0085 (3)	0.0147 (3)	0.0064 (3)

### 2,3-Dimethoxybenzaldehyde (23DMBz) Geometric parameters (Å, º)

**Table:**

O01—C07	1.3750 (8)	C06—H06	0.9500
O01—C11	1.4383 (10)	C08—H08	0.9500
O02—C05	1.3601 (8)	C09—C10	1.3960 (11)
O02—C12	1.4284 (9)	C09—H09	0.9500
O03—C08	1.2175 (9)	C10—H10	0.9500
C04—C07	1.3945 (9)	C11—H11A	0.9800
C04—C06	1.4029 (10)	C11—H11B	0.9800
C04—C08	1.4767 (10)	C11—H11C	0.9800
C05—C09	1.3901 (10)	C12—H12A	0.9800
C05—C07	1.4085 (9)	C12—H12B	0.9800
C06—C10	1.3807 (12)	C12—H12C	0.9800
			
C07—O01—C11	112.47 (6)	C05—C09—H09	120.0
C05—O02—C12	117.16 (6)	C10—C09—H09	120.0
C07—C04—C06	119.94 (7)	C06—C10—C09	120.82 (7)
C07—C04—C08	120.08 (6)	C06—C10—H10	119.6
C06—C04—C08	119.98 (6)	C09—C10—H10	119.6
O02—C05—C09	124.88 (6)	O01—C11—H11A	109.5
O02—C05—C07	115.51 (6)	O01—C11—H11B	109.5
C09—C05—C07	119.59 (6)	H11A—C11—H11B	109.5
C10—C06—C04	119.71 (7)	O01—C11—H11C	109.5
C10—C06—H06	120.1	H11A—C11—H11C	109.5
C04—C06—H06	120.1	H11B—C11—H11C	109.5
O01—C07—C04	120.23 (6)	O02—C12—H12A	109.5
O01—C07—C05	119.76 (6)	O02—C12—H12B	109.5
C04—C07—C05	119.96 (6)	H12A—C12—H12B	109.5
O03—C08—C04	123.28 (8)	O02—C12—H12C	109.5
O03—C08—H08	118.4	H12A—C12—H12C	109.5
C04—C08—H08	118.4	H12B—C12—H12C	109.5
C05—C09—C10	119.98 (7)		
			
C12—O02—C05—C09	2.55 (11)	O02—C05—C07—O01	−1.05 (9)
C12—O02—C05—C07	−178.75 (6)	C09—C05—C07—O01	177.73 (6)
C07—C04—C06—C10	0.13 (11)	O02—C05—C07—C04	−178.28 (6)
C08—C04—C06—C10	−179.23 (7)	C09—C05—C07—C04	0.51 (10)
C11—O01—C07—C04	−108.70 (7)	C07—C04—C08—O03	−175.45 (7)
C11—O01—C07—C05	74.09 (8)	C06—C04—C08—O03	3.90 (11)
C06—C04—C07—O01	−177.61 (6)	O02—C05—C09—C10	178.31 (7)
C08—C04—C07—O01	1.75 (10)	C07—C05—C09—C10	−0.34 (11)
C06—C04—C07—C05	−0.40 (10)	C04—C06—C10—C09	0.03 (11)
C08—C04—C07—C05	178.96 (6)	C05—C09—C10—C06	0.08 (11)

### 2,4-Dimethoxybenzaldehyde (24DMBz) Crystal data

**Table:**

C_9_H_10_O_3_	*D*_x_ = 1.374 Mg m^−^^3^
*M**_r_* = 166.17	Melting point: 341 K
Monoclinic, *P*2_1_/*c*	Mo *K*α radiation, λ = 0.71073 Å
*a* = 15.1575 (8) Å	Cell parameters from 9286 reflections
*b* = 3.9638 (2) Å	θ = 2.8–30.5°
*c* = 14.6181 (8) Å	µ = 0.10 mm^−^^1^
β = 113.8388 (19)°	*T* = 150 K
*V* = 803.35 (7) Å^3^	Block, colourless
*Z* = 4	0.50 × 0.43 × 0.40 mm
*F*(000) = 352	

### 2,4-Dimethoxybenzaldehyde (24DMBz) Data collection

**Table:**

Bruker D8 Quest APEX3 diffractometer	2461 independent reflections
Radiation source: sealed tube	2171 reflections with *I* > 2σ(*I*)
Graphite monochromator	*R*_int_ = 0.020
Detector resolution: 10.4 pixels mm^-1^	θ_max_ = 30.5°, θ_min_ = 2.8°
φ and ω scans	*h* = −21→21
Absorption correction: multi-scan (SADABS; Krause et al., 2015)	*k* = −5→5
*T*_min_ = 0.685, *T*_max_ = 0.746	*l* = −20→19
15236 measured reflections	

### 2,4-Dimethoxybenzaldehyde (24DMBz) Refinement

**Table:**

Refinement on *F*^2^	Primary atom site location: structure-invariant direct methods
Least-squares matrix: full	Secondary atom site location: difference Fourier map
*R*[*F*^2^ > 2σ(*F*^2^)] = 0.039	Hydrogen site location: inferred from neighbouring sites
*wR*(*F*^2^) = 0.117	H-atom parameters constrained
*S* = 1.03	*w* = 1/[σ^2^(*F*_o_^2^) + (0.0714*P*)^2^ + 0.196*P*] where *P* = (*F*_o_^2^ + 2*F*_c_^2^)/3
2461 reflections	(Δ/σ)_max_ < 0.001
111 parameters	Δρ_max_ = 0.40 e Å^−^^3^
0 restraints	Δρ_min_ = −0.24 e Å^−^^3^

### 2,4-Dimethoxybenzaldehyde (24DMBz) Special details

**Table:**

Geometry. All esds (except the esd in the dihedral angle between two l.s. planes) are estimated using the full covariance matrix. The cell esds are taken into account individually in the estimation of esds in distances, angles and torsion angles; correlations between esds in cell parameters are only used when they are defined by crystal symmetry. An approximate (isotropic) treatment of cell esds is used for estimating esds involving l.s. planes.

### 2,4-Dimethoxybenzaldehyde (24DMBz) Fractional atomic coordinates and isotropic or equivalent isotropic displacement parameters (Å^2^)

**Table:**

	*x*	*y*	*z*	*U*_iso_*/*U*_eq_	
O01	0.08102 (5)	0.65429 (18)	0.10801 (5)	0.02325 (16)	
O02	0.39771 (5)	0.76391 (19)	0.37610 (5)	0.02571 (17)	
O03	0.31680 (6)	0.2357 (2)	0.56154 (5)	0.0358 (2)	
C04	0.30419 (6)	0.6579 (2)	0.33618 (6)	0.01822 (17)	
C05	0.27290 (6)	0.4771 (2)	0.40045 (6)	0.01952 (18)	
C06	0.14709 (6)	0.6063 (2)	0.20292 (6)	0.01766 (17)	
C07	0.24207 (6)	0.7210 (2)	0.23720 (6)	0.01809 (17)	
H07	0.263957	0.839754	0.193881	0.022*	
C08	0.11380 (6)	0.4292 (2)	0.26563 (6)	0.01980 (18)	
H08	0.048785	0.354354	0.241536	0.024*	
C09	0.17672 (6)	0.3653 (2)	0.36266 (6)	0.02020 (18)	
H09	0.154643	0.242605	0.405167	0.024*	
C10	0.11345 (7)	0.8119 (2)	0.03878 (7)	0.02355 (19)	
H10A	0.133339	1.044263	0.060154	0.035*	
H10B	0.060848	0.813520	−0.028047	0.035*	
H10C	0.168300	0.685728	0.036939	0.035*	
C11	0.33908 (7)	0.3954 (3)	0.50273 (7)	0.0272 (2)	
H11	0.403821	0.471787	0.524941	0.033*	
C12	0.43374 (7)	0.9262 (3)	0.31058 (8)	0.0276 (2)	
H12A	0.425511	0.776125	0.254432	0.041*	
H12B	0.502361	0.977611	0.347363	0.041*	
H12C	0.397996	1.135995	0.285113	0.041*	

### 2,4-Dimethoxybenzaldehyde (24DMBz) Atomic displacement parameters (Å^2^)

**Table:**

	*U*^11^	*U*^22^	*U*^33^	*U*^12^	*U*^13^	*U*^23^
O01	0.0204 (3)	0.0285 (3)	0.0187 (3)	−0.0020 (2)	0.0057 (2)	0.0016 (2)
O02	0.0180 (3)	0.0327 (4)	0.0249 (3)	−0.0049 (3)	0.0071 (2)	0.0042 (3)
O03	0.0340 (4)	0.0489 (5)	0.0239 (3)	−0.0020 (3)	0.0111 (3)	0.0108 (3)
C04	0.0169 (3)	0.0182 (4)	0.0204 (4)	0.0001 (3)	0.0084 (3)	−0.0010 (3)
C05	0.0211 (4)	0.0201 (4)	0.0186 (4)	0.0008 (3)	0.0093 (3)	0.0001 (3)
C06	0.0189 (4)	0.0163 (3)	0.0181 (3)	0.0013 (3)	0.0077 (3)	−0.0022 (3)
C07	0.0194 (4)	0.0176 (3)	0.0194 (4)	0.0003 (3)	0.0100 (3)	−0.0001 (3)
C08	0.0191 (4)	0.0194 (4)	0.0229 (4)	−0.0016 (3)	0.0106 (3)	−0.0018 (3)
C09	0.0229 (4)	0.0198 (4)	0.0218 (4)	−0.0003 (3)	0.0131 (3)	0.0000 (3)
C10	0.0269 (4)	0.0249 (4)	0.0188 (4)	−0.0002 (3)	0.0092 (3)	0.0011 (3)
C11	0.0251 (4)	0.0335 (5)	0.0215 (4)	−0.0011 (4)	0.0078 (3)	0.0034 (3)
C12	0.0215 (4)	0.0296 (5)	0.0337 (5)	−0.0017 (3)	0.0134 (4)	0.0068 (4)

### 2,4-Dimethoxybenzaldehyde (24DMBz) Geometric parameters (Å, º)

**Table:**

O01—C06	1.3567 (10)	C07—H07	0.9500
O01—C10	1.4347 (11)	C08—C09	1.3756 (12)
O02—C04	1.3629 (10)	C08—H08	0.9500
O02—C12	1.4326 (11)	C09—H09	0.9500
O03—C11	1.2201 (12)	C10—H10A	0.9800
C04—C07	1.3934 (11)	C10—H10B	0.9800
C04—C05	1.4077 (11)	C10—H10C	0.9800
C05—C09	1.4057 (12)	C11—H11	0.9500
C05—C11	1.4608 (12)	C12—H12A	0.9800
C06—C07	1.3954 (11)	C12—H12B	0.9800
C06—C08	1.4007 (11)	C12—H12C	0.9800
			
C06—O01—C10	117.43 (7)	C08—C09—H09	119.2
C04—O02—C12	117.63 (7)	C05—C09—H09	119.2
O02—C04—C07	122.59 (7)	O01—C10—H10A	109.5
O02—C04—C05	116.35 (7)	O01—C10—H10B	109.5
C07—C04—C05	121.05 (7)	H10A—C10—H10B	109.5
C09—C05—C04	118.29 (7)	O01—C10—H10C	109.5
C09—C05—C11	120.43 (8)	H10A—C10—H10C	109.5
C04—C05—C11	121.24 (8)	H10B—C10—H10C	109.5
O01—C06—C07	123.34 (8)	O03—C11—C05	124.40 (9)
O01—C06—C08	115.29 (7)	O03—C11—H11	117.8
C07—C06—C08	121.37 (8)	C05—C11—H11	117.8
C04—C07—C06	118.72 (8)	O02—C12—H12A	109.5
C04—C07—H07	120.6	O02—C12—H12B	109.5
C06—C07—H07	120.6	H12A—C12—H12B	109.5
C09—C08—C06	118.95 (8)	O02—C12—H12C	109.5
C09—C08—H08	120.5	H12A—C12—H12C	109.5
C06—C08—H08	120.5	H12B—C12—H12C	109.5
C08—C09—C05	121.60 (8)		
			
C12—O02—C04—C07	−4.23 (12)	O01—C06—C07—C04	−179.94 (7)
C12—O02—C04—C05	175.40 (8)	C08—C06—C07—C04	−0.36 (12)
O02—C04—C05—C09	179.48 (8)	O01—C06—C08—C09	179.01 (7)
C07—C04—C05—C09	−0.88 (13)	C07—C06—C08—C09	−0.60 (12)
O02—C04—C05—C11	−2.82 (13)	C06—C08—C09—C05	0.83 (13)
C07—C04—C05—C11	176.82 (8)	C04—C05—C09—C08	−0.11 (13)
C10—O01—C06—C07	4.17 (12)	C11—C05—C09—C08	−177.83 (8)
C10—O01—C06—C08	−175.43 (7)	C09—C05—C11—O03	−1.27 (16)
O02—C04—C07—C06	−179.28 (8)	C04—C05—C11—O03	−178.93 (10)
C05—C04—C07—C06	1.10 (12)		

### 2,5-Dimethoxybenzaldehyde (25DMBz) Crystal data

**Table:**

C_9_H_10_O_3_	*D*_x_ = 1.384 Mg m^−^^3^
*M**_r_* = 166.17	Melting point: 321 K
Monoclinic, *P*2_1_/*n*	Mo *K*α radiation, λ = 0.71073 Å
*a* = 3.8780 (3) Å	Cell parameters from 9955 reflections
*b* = 11.5513 (7) Å	θ = 2.3–36.2°
*c* = 17.8153 (12) Å	µ = 0.10 mm^−^^1^
β = 91.808 (2)°	*T* = 150 K
*V* = 797.66 (10) Å^3^	Needle, colourless
*Z* = 4	0.74 × 0.38 × 0.13 mm
*F*(000) = 352	

### 2,5-Dimethoxybenzaldehyde (25DMBz) Data collection

**Table:**

Bruker D8 Quest APEX3 diffractometer	3873 independent reflections
Radiation source: sealed tube	3276 reflections with *I* > 2σ(*I*)
Graphite monochromator	*R*_int_ = 0.024
Detector resolution: 10.4 pixels mm^-1^	θ_max_ = 36.4°, θ_min_ = 2.1°
φ and ω scans	*h* = −6→6
Absorption correction: multi-scan (SADABS; Krause et al., 2015)	*k* = −14→19
*T*_min_ = 0.705, *T*_max_ = 0.747	*l* = −29→29
30235 measured reflections	

### 2,5-Dimethoxybenzaldehyde (25DMBz) Refinement

**Table:**

Refinement on *F*^2^	Primary atom site location: structure-invariant direct methods
Least-squares matrix: full	Secondary atom site location: difference Fourier map
*R*[*F*^2^ > 2σ(*F*^2^)] = 0.039	Hydrogen site location: inferred from neighbouring sites
*wR*(*F*^2^) = 0.124	H-atom parameters constrained
*S* = 1.02	*w* = 1/[σ^2^(*F*_o_^2^) + (0.0691*P*)^2^ + 0.1755*P*] where *P* = (*F*_o_^2^ + 2*F*_c_^2^)/3
3873 reflections	(Δ/σ)_max_ = 0.001
111 parameters	Δρ_max_ = 0.54 e Å^−^^3^
0 restraints	Δρ_min_ = −0.22 e Å^−^^3^

### 2,5-Dimethoxybenzaldehyde (25DMBz) Special details

**Table:**

Geometry. All esds (except the esd in the dihedral angle between two l.s. planes) are estimated using the full covariance matrix. The cell esds are taken into account individually in the estimation of esds in distances, angles and torsion angles; correlations between esds in cell parameters are only used when they are defined by crystal symmetry. An approximate (isotropic) treatment of cell esds is used for estimating esds involving l.s. planes.

### 2,5-Dimethoxybenzaldehyde (25DMBz) Fractional atomic coordinates and isotropic or equivalent isotropic displacement parameters (Å^2^)

**Table:**

	*x*	*y*	*z*	*U*_iso_*/*U*_eq_	
O01	0.70027 (15)	0.37815 (5)	0.56706 (3)	0.02308 (11)	
O02	0.32551 (16)	0.55079 (5)	0.84287 (3)	0.02348 (12)	
O03	0.81653 (19)	0.71583 (5)	0.60790 (3)	0.02970 (14)	
C04	0.64196 (16)	0.53211 (5)	0.65291 (3)	0.01623 (11)	
C05	0.54777 (16)	0.57451 (5)	0.72267 (3)	0.01719 (11)	
H05	0.580616	0.654118	0.734217	0.021*	
C06	0.44894 (18)	0.34122 (6)	0.68849 (4)	0.01849 (12)	
H06	0.413247	0.261704	0.677056	0.022*	
C07	0.40630 (16)	0.50128 (5)	0.77542 (3)	0.01656 (11)	
C08	0.35490 (17)	0.38467 (6)	0.75789 (4)	0.01814 (12)	
H08	0.255150	0.334576	0.793501	0.022*	
C09	0.59531 (16)	0.41403 (5)	0.63571 (3)	0.01655 (11)	
C10	0.79366 (19)	0.61160 (6)	0.59830 (4)	0.02189 (13)	
H10	0.878085	0.579933	0.553220	0.026*	
C11	0.6662 (2)	0.25766 (6)	0.55053 (4)	0.02393 (14)	
H11A	0.787349	0.212440	0.589721	0.036*	
H11B	0.766517	0.241337	0.501841	0.036*	
H11C	0.421362	0.236493	0.548665	0.036*	
C12	0.1987 (2)	0.47519 (7)	0.89872 (4)	0.02583 (15)	
H12A	−0.020111	0.441090	0.880789	0.039*	
H12B	0.161901	0.518945	0.944911	0.039*	
H12C	0.367146	0.413466	0.908934	0.039*	

### 2,5-Dimethoxybenzaldehyde (25DMBz) Atomic displacement parameters (Å^2^)

**Table:**

	*U*^11^	*U*^22^	*U*^33^	*U*^12^	*U*^13^	*U*^23^
O01	0.0330 (3)	0.0181 (2)	0.0185 (2)	−0.00401 (19)	0.00611 (18)	−0.00154 (16)
O02	0.0348 (3)	0.0174 (2)	0.0187 (2)	0.00073 (19)	0.00818 (19)	0.00082 (16)
O03	0.0450 (3)	0.0186 (2)	0.0256 (3)	−0.0097 (2)	0.0032 (2)	0.00350 (18)
C04	0.0178 (2)	0.0145 (2)	0.0164 (2)	−0.00141 (18)	0.00017 (18)	0.00262 (18)
C05	0.0197 (2)	0.0141 (2)	0.0178 (2)	−0.00057 (19)	0.00054 (19)	0.00188 (18)
C06	0.0222 (3)	0.0146 (2)	0.0186 (2)	−0.00275 (19)	0.0011 (2)	0.00149 (18)
C07	0.0179 (2)	0.0156 (2)	0.0163 (2)	0.00086 (19)	0.00157 (18)	0.00161 (18)
C08	0.0205 (3)	0.0158 (2)	0.0183 (2)	−0.00184 (19)	0.00202 (19)	0.00261 (18)
C09	0.0182 (2)	0.0155 (2)	0.0160 (2)	−0.00112 (19)	0.00031 (18)	0.00083 (18)
C10	0.0277 (3)	0.0191 (3)	0.0189 (3)	−0.0051 (2)	0.0018 (2)	0.0032 (2)
C11	0.0306 (3)	0.0194 (3)	0.0219 (3)	−0.0011 (2)	0.0020 (2)	−0.0034 (2)
C12	0.0322 (4)	0.0232 (3)	0.0227 (3)	0.0033 (3)	0.0109 (3)	0.0042 (2)

### 2,5-Dimethoxybenzaldehyde (25DMBz) Geometric parameters (Å, º)

**Table:**

O01—C09	1.3655 (8)	C06—C09	1.3954 (9)
O01—C11	1.4280 (9)	C06—H06	0.9500
O02—C07	1.3757 (8)	C07—C08	1.3957 (9)
O02—C12	1.4232 (9)	C08—H08	0.9500
O03—C10	1.2189 (9)	C10—H10	0.9500
C04—C05	1.3952 (9)	C11—H11A	0.9800
C04—C09	1.4083 (9)	C11—H11B	0.9800
C04—C10	1.4738 (9)	C11—H11C	0.9800
C05—C07	1.3901 (9)	C12—H12A	0.9800
C05—H05	0.9500	C12—H12B	0.9800
C06—C08	1.3936 (9)	C12—H12C	0.9800
			
C09—O01—C11	116.90 (5)	O01—C09—C04	116.67 (5)
C07—O02—C12	116.67 (6)	C06—C09—C04	119.30 (6)
C05—C04—C09	119.89 (6)	O03—C10—C04	123.47 (7)
C05—C04—C10	119.37 (6)	O03—C10—H10	118.3
C09—C04—C10	120.73 (6)	C04—C10—H10	118.3
C07—C05—C04	120.57 (6)	O01—C11—H11A	109.5
C07—C05—H05	119.7	O01—C11—H11B	109.5
C04—C05—H05	119.7	H11A—C11—H11B	109.5
C08—C06—C09	120.27 (6)	O01—C11—H11C	109.5
C08—C06—H06	119.9	H11A—C11—H11C	109.5
C09—C06—H06	119.9	H11B—C11—H11C	109.5
O02—C07—C05	116.31 (6)	O02—C12—H12A	109.5
O02—C07—C08	124.18 (6)	O02—C12—H12B	109.5
C05—C07—C08	119.51 (6)	H12A—C12—H12B	109.5
C06—C08—C07	120.44 (6)	O02—C12—H12C	109.5
C06—C08—H08	119.8	H12A—C12—H12C	109.5
C07—C08—H08	119.8	H12B—C12—H12C	109.5
O01—C09—C06	124.03 (6)		
			
C09—C04—C05—C07	−0.32 (9)	C11—O01—C09—C04	177.58 (6)
C10—C04—C05—C07	−179.56 (6)	C08—C06—C09—O01	178.78 (6)
C12—O02—C07—C05	−176.57 (6)	C08—C06—C09—C04	−0.97 (10)
C12—O02—C07—C08	3.41 (10)	C05—C04—C09—O01	−178.60 (6)
C04—C05—C07—O02	179.25 (6)	C10—C04—C09—O01	0.63 (9)
C04—C05—C07—C08	−0.72 (10)	C05—C04—C09—C06	1.16 (9)
C09—C06—C08—C07	−0.07 (10)	C10—C04—C09—C06	−179.60 (6)
O02—C07—C08—C06	−179.05 (6)	C05—C04—C10—O03	−6.37 (11)
C05—C07—C08—C06	0.92 (10)	C09—C04—C10—O03	174.39 (7)
C11—O01—C09—C06	−2.17 (10)		

### 3,5-Dimethoxybenzaldehyde (35DMBz) Crystal data

**Table:**

C_9_H_10_O_3_	*D*_x_ = 1.346 Mg m^−^^3^
*M**_r_* = 166.17	Melting point: 319 K
Monoclinic, *P*2_1_/*c*	Mo *K*α radiation, λ = 0.71073 Å
*a* = 11.7602 (5) Å	Cell parameters from 9794 reflections
*b* = 13.8957 (6) Å	θ = 2.5–36.4°
*c* = 11.4352 (5) Å	µ = 0.10 mm^−^^1^
β = 118.642 (2)°	*T* = 150 K
*V* = 1640.03 (13) Å^3^	Block, colourless
*Z* = 8	0.50 × 0.43 × 0.40 mm
*F*(000) = 704	

### 3,5-Dimethoxybenzaldehyde (35DMBz) Data collection

**Table:**

Bruker D8 Quest APEX3 diffractometer	7976 independent reflections
Radiation source: sealed tube	6730 reflections with *I* > 2σ(*I*)
Graphite monochromator	*R*_int_ = 0.030
Detector resolution: 10.4 pixels mm^-1^	θ_max_ = 36.5°, θ_min_ = 2.5°
φ and ω scans	*h* = −19→19
Absorption correction: multi-scan (SADABS; Krause et al., 2015)	*k* = −23→22
*T*_min_ = 0.703, *T*_max_ = 0.747	*l* = −19→19
53075 measured reflections	

### 3,5-Dimethoxybenzaldehyde (35DMBz) Refinement

**Table:**

Refinement on *F*^2^	Primary atom site location: structure-invariant direct methods
Least-squares matrix: full	Secondary atom site location: difference Fourier map
*R*[*F*^2^ > 2σ(*F*^2^)] = 0.042	Hydrogen site location: inferred from neighbouring sites
*wR*(*F*^2^) = 0.126	H-atom parameters constrained
*S* = 1.05	*w* = 1/[σ^2^(*F*_o_^2^) + (0.0699*P*)^2^ + 0.3147*P*] where *P* = (*F*_o_^2^ + 2*F*_c_^2^)/3
7976 reflections	(Δ/σ)_max_ = 0.001
222 parameters	Δρ_max_ = 0.48 e Å^−^^3^
0 restraints	Δρ_min_ = −0.25 e Å^−^^3^

### 3,5-Dimethoxybenzaldehyde (35DMBz) Special details

**Table:**

Geometry. All esds (except the esd in the dihedral angle between two l.s. planes) are estimated using the full covariance matrix. The cell esds are taken into account individually in the estimation of esds in distances, angles and torsion angles; correlations between esds in cell parameters are only used when they are defined by crystal symmetry. An approximate (isotropic) treatment of cell esds is used for estimating esds involving l.s. planes.
Refinement. Refined as a two-component twin.

### 3,5-Dimethoxybenzaldehyde (35DMBz) Fractional atomic coordinates and isotropic or equivalent isotropic displacement parameters (Å^2^)

**Table:**

	*x*	*y*	*z*	*U*_iso_*/*U*_eq_	
O01	0.10858 (7)	0.50152 (5)	0.16802 (7)	0.02241 (12)	
O02	−0.14028 (7)	0.31769 (5)	0.35652 (7)	0.02335 (13)	
O03	−0.07159 (7)	0.74381 (5)	0.32465 (7)	0.02290 (13)	
O04	0.38859 (7)	0.67098 (5)	0.07120 (6)	0.02131 (12)	
O05	0.56249 (7)	0.91448 (4)	0.40230 (6)	0.02143 (12)	
O06	0.64980 (9)	0.48884 (5)	0.50429 (8)	0.03135 (16)	
C07	0.01767 (7)	0.62606 (5)	0.24757 (8)	0.01641 (12)	
H07	0.055272	0.676140	0.220862	0.020*	
C08	−0.11303 (7)	0.57541 (5)	0.34891 (7)	0.01585 (12)	
H08	−0.163797	0.590389	0.390752	0.019*	
C09	0.03688 (7)	0.53026 (6)	0.22608 (8)	0.01607 (12)	
C10	0.46028 (7)	0.69980 (6)	0.20008 (8)	0.01623 (12)	
C11	−0.09212 (7)	0.47970 (5)	0.32589 (7)	0.01525 (12)	
C12	−0.05762 (7)	0.64810 (5)	0.30898 (8)	0.01594 (12)	
C13	0.60966 (8)	0.74637 (6)	0.47010 (8)	0.01768 (13)	
H13	0.659966	0.761890	0.561868	0.021*	
C14	−0.01837 (8)	0.45589 (6)	0.26492 (8)	0.01680 (12)	
H14	−0.005616	0.390505	0.249828	0.020*	
C15	0.55203 (7)	0.81845 (5)	0.37503 (7)	0.01604 (12)	
C16	0.47689 (7)	0.79570 (6)	0.23988 (7)	0.01637 (12)	
H16	0.437535	0.845382	0.175813	0.020*	
C17	0.65223 (10)	0.57391 (7)	0.52876 (9)	0.02399 (16)	
H17	0.696151	0.592882	0.619685	0.029*	
C18	0.51868 (8)	0.62611 (6)	0.29356 (8)	0.01800 (13)	
H18	0.508552	0.560727	0.266097	0.022*	
C19	0.59175 (8)	0.65038 (6)	0.42732 (8)	0.01768 (13)	
C20	−0.14979 (8)	0.40334 (6)	0.37109 (8)	0.01879 (13)	
H20	−0.197577	0.422663	0.414534	0.023*	
C21	−0.14813 (10)	0.76918 (6)	0.38642 (10)	0.02406 (16)	
H21A	−0.235885	0.743391	0.333753	0.036*	
H21B	−0.152196	0.839410	0.391306	0.036*	
H21C	−0.108775	0.742127	0.476544	0.036*	
C22	0.30925 (9)	0.74150 (7)	−0.02458 (8)	0.02306 (15)	
H22A	0.251864	0.771607	0.004783	0.035*	
H22B	0.257053	0.710378	−0.111097	0.035*	
H22C	0.364524	0.790773	−0.032972	0.035*	
C23	0.64453 (9)	0.94220 (6)	0.53742 (8)	0.02092 (14)	
H23A	0.611474	0.914527	0.594092	0.031*	
H23B	0.645839	1.012531	0.544393	0.031*	
H23C	0.732586	0.918592	0.566549	0.031*	
C24	0.17317 (10)	0.57404 (7)	0.13303 (11)	0.02795 (19)	
H24A	0.108750	0.616172	0.064624	0.042*	
H24B	0.226038	0.543502	0.098340	0.042*	
H24C	0.228988	0.612158	0.212125	0.042*	

### 3,5-Dimethoxybenzaldehyde (35DMBz) Atomic displacement parameters (Å^2^)

**Table:**

	*U*^11^	*U*^22^	*U*^33^	*U*^12^	*U*^13^	*U*^23^
O01	0.0291 (3)	0.0174 (3)	0.0313 (3)	−0.0012 (2)	0.0229 (3)	−0.0018 (2)
O02	0.0322 (3)	0.0155 (3)	0.0262 (3)	−0.0037 (2)	0.0170 (3)	−0.0008 (2)
O03	0.0310 (3)	0.0124 (2)	0.0347 (3)	0.0003 (2)	0.0233 (3)	−0.0003 (2)
O04	0.0257 (3)	0.0174 (3)	0.0159 (2)	0.0009 (2)	0.0060 (2)	−0.00314 (19)
O05	0.0276 (3)	0.0133 (2)	0.0164 (2)	0.0012 (2)	0.0050 (2)	−0.00162 (18)
O06	0.0421 (4)	0.0170 (3)	0.0329 (4)	0.0070 (3)	0.0164 (3)	0.0048 (3)
C07	0.0178 (3)	0.0140 (3)	0.0191 (3)	−0.0010 (2)	0.0103 (2)	0.0003 (2)
C08	0.0182 (3)	0.0144 (3)	0.0166 (3)	−0.0007 (2)	0.0097 (2)	0.0002 (2)
C09	0.0173 (3)	0.0151 (3)	0.0179 (3)	−0.0006 (2)	0.0101 (2)	−0.0004 (2)
C10	0.0170 (3)	0.0156 (3)	0.0160 (3)	0.0003 (2)	0.0078 (2)	−0.0015 (2)
C11	0.0176 (3)	0.0138 (3)	0.0148 (3)	−0.0011 (2)	0.0081 (2)	0.0007 (2)
C12	0.0181 (3)	0.0128 (3)	0.0179 (3)	−0.0005 (2)	0.0094 (2)	−0.0001 (2)
C13	0.0202 (3)	0.0162 (3)	0.0158 (3)	0.0023 (2)	0.0079 (2)	0.0009 (2)
C14	0.0201 (3)	0.0138 (3)	0.0185 (3)	−0.0007 (2)	0.0109 (2)	−0.0001 (2)
C15	0.0174 (3)	0.0137 (3)	0.0163 (3)	0.0012 (2)	0.0075 (2)	−0.0006 (2)
C16	0.0180 (3)	0.0144 (3)	0.0154 (3)	0.0011 (2)	0.0070 (2)	−0.0005 (2)
C17	0.0305 (4)	0.0185 (3)	0.0221 (3)	0.0059 (3)	0.0119 (3)	0.0045 (3)
C18	0.0210 (3)	0.0144 (3)	0.0193 (3)	0.0011 (2)	0.0102 (3)	−0.0002 (2)
C19	0.0206 (3)	0.0148 (3)	0.0183 (3)	0.0027 (2)	0.0098 (3)	0.0019 (2)
C20	0.0232 (3)	0.0161 (3)	0.0197 (3)	−0.0030 (2)	0.0125 (3)	0.0002 (2)
C21	0.0301 (4)	0.0173 (3)	0.0324 (4)	0.0023 (3)	0.0212 (4)	−0.0013 (3)
C22	0.0249 (4)	0.0231 (4)	0.0165 (3)	0.0040 (3)	0.0062 (3)	−0.0012 (3)
C23	0.0248 (3)	0.0175 (3)	0.0170 (3)	−0.0018 (3)	0.0072 (3)	−0.0029 (2)
C24	0.0334 (4)	0.0236 (4)	0.0400 (5)	−0.0051 (3)	0.0281 (4)	−0.0032 (4)

### 3,5-Dimethoxybenzaldehyde (35DMBz) Geometric parameters (Å, º)

**Table:**

O01—C09	1.3594 (10)	C13—C19	1.4014 (11)
O01—C24	1.4297 (11)	C13—H13	0.9500
O02—C20	1.2144 (10)	C14—H14	0.9500
O03—C12	1.3624 (10)	C15—C16	1.4000 (10)
O03—C21	1.4292 (11)	C16—H16	0.9500
O04—C10	1.3609 (10)	C17—C19	1.4797 (12)
O04—C22	1.4321 (11)	C17—H17	0.9500
O05—C15	1.3623 (9)	C18—C19	1.3898 (11)
O05—C23	1.4275 (10)	C18—H18	0.9500
O06—C17	1.2120 (12)	C20—H20	0.9500
C07—C09	1.3918 (11)	C21—H21A	0.9800
C07—C12	1.4025 (11)	C21—H21B	0.9800
C07—H07	0.9500	C21—H21C	0.9800
C08—C12	1.3923 (11)	C22—H22A	0.9800
C08—C11	1.4003 (11)	C22—H22B	0.9800
C08—H08	0.9500	C22—H22C	0.9800
C09—C14	1.4014 (11)	C23—H23A	0.9800
C10—C16	1.3914 (11)	C23—H23B	0.9800
C10—C18	1.3996 (11)	C23—H23C	0.9800
C11—C14	1.3884 (11)	C24—H24A	0.9800
C11—C20	1.4795 (11)	C24—H24B	0.9800
C13—C15	1.3922 (11)	C24—H24C	0.9800
			
C09—O01—C24	117.83 (7)	O06—C17—H17	117.6
C12—O03—C21	116.74 (7)	C19—C17—H17	117.6
C10—O04—C22	117.72 (7)	C19—C18—C10	118.77 (7)
C15—O05—C23	116.88 (6)	C19—C18—H18	120.6
C09—C07—C12	119.48 (7)	C10—C18—H18	120.6
C09—C07—H07	120.3	C18—C19—C13	121.69 (7)
C12—C07—H07	120.3	C18—C19—C17	119.99 (7)
C12—C08—C11	118.39 (7)	C13—C19—C17	118.32 (7)
C12—C08—H08	120.8	O02—C20—C11	124.56 (8)
C11—C08—H08	120.8	O02—C20—H20	117.7
O01—C09—C07	123.95 (7)	C11—C20—H20	117.7
O01—C09—C14	115.37 (7)	O03—C21—H21A	109.5
C07—C09—C14	120.68 (7)	O03—C21—H21B	109.5
O04—C10—C16	123.58 (7)	H21A—C21—H21B	109.5
O04—C10—C18	115.74 (7)	O03—C21—H21C	109.5
C16—C10—C18	120.68 (7)	H21A—C21—H21C	109.5
C14—C11—C08	121.94 (7)	H21B—C21—H21C	109.5
C14—C11—C20	120.39 (7)	O04—C22—H22A	109.5
C08—C11—C20	117.66 (7)	O04—C22—H22B	109.5
O03—C12—C08	124.06 (7)	H22A—C22—H22B	109.5
O03—C12—C07	115.08 (7)	O04—C22—H22C	109.5
C08—C12—C07	120.86 (7)	H22A—C22—H22C	109.5
C15—C13—C19	118.47 (7)	H22B—C22—H22C	109.5
C15—C13—H13	120.8	O05—C23—H23A	109.5
C19—C13—H13	120.8	O05—C23—H23B	109.5
C11—C14—C09	118.66 (7)	H23A—C23—H23B	109.5
C11—C14—H14	120.7	O05—C23—H23C	109.5
C09—C14—H14	120.7	H23A—C23—H23C	109.5
O05—C15—C13	124.69 (7)	H23B—C23—H23C	109.5
O05—C15—C16	114.44 (7)	O01—C24—H24A	109.5
C13—C15—C16	120.86 (7)	O01—C24—H24B	109.5
C10—C16—C15	119.52 (7)	H24A—C24—H24B	109.5
C10—C16—H16	120.2	O01—C24—H24C	109.5
C15—C16—H16	120.2	H24A—C24—H24C	109.5
O06—C17—C19	124.76 (9)	H24B—C24—H24C	109.5
			
C24—O01—C09—C07	3.19 (13)	C23—O05—C15—C13	3.63 (12)
C24—O01—C09—C14	−176.62 (8)	C23—O05—C15—C16	−176.23 (7)
C12—C07—C09—O01	−179.69 (7)	C19—C13—C15—O05	−179.27 (8)
C12—C07—C09—C14	0.11 (12)	C19—C13—C15—C16	0.58 (12)
C22—O04—C10—C16	−10.72 (12)	O04—C10—C16—C15	179.72 (7)
C22—O04—C10—C18	169.48 (8)	C18—C10—C16—C15	−0.50 (12)
C12—C08—C11—C14	−0.02 (11)	O05—C15—C16—C10	179.48 (7)
C12—C08—C11—C20	178.95 (7)	C13—C15—C16—C10	−0.38 (12)
C21—O03—C12—C08	0.13 (12)	O04—C10—C18—C19	−179.06 (7)
C21—O03—C12—C07	179.77 (8)	C16—C10—C18—C19	1.13 (12)
C11—C08—C12—O03	179.42 (7)	C10—C18—C19—C13	−0.93 (12)
C11—C08—C12—C07	−0.20 (11)	C10—C18—C19—C17	178.64 (8)
C09—C07—C12—O03	−179.50 (7)	C15—C13—C19—C18	0.09 (12)
C09—C07—C12—C08	0.15 (12)	C15—C13—C19—C17	−179.49 (8)
C08—C11—C14—C09	0.28 (11)	O06—C17—C19—C18	4.47 (15)
C20—C11—C14—C09	−178.66 (7)	O06—C17—C19—C13	−175.94 (10)
O01—C09—C14—C11	179.50 (7)	C14—C11—C20—O02	−1.69 (13)
C07—C09—C14—C11	−0.32 (12)	C08—C11—C20—O02	179.33 (8)
